# Saturated fatty acids induce development of both metabolic syndrome and osteoarthritis in rats

**DOI:** 10.1038/srep46457

**Published:** 2017-04-18

**Authors:** Sunderajhan Sekar, Siti Raihanah Shafie, Indira Prasadam, Ross Crawford, Sunil K. Panchal, Lindsay Brown, Yin Xiao

**Affiliations:** 1 Institute of Health and Biomedical Innovation, School of Chemistry, Physics, Mechanical Engineering, Queensland University of Technology, Brisbane, Australia; 2 Institute for Agriculture and the Environment, and School of Health and Wellbeing, University of Southern Queensland, Toowoomba, Queensland, Australia; 3 The Prince Charles Hospital, Orthopaedic Department, Brisbane, Australia

## Abstract

The predominant saturated fatty acids (SFA) in human diets are lauric acid (LA, C12:0), myristic acid (MA, C14:0), palmitic acid (PA, C16:0) and stearic acid (SA, C18:0). The aim of this study was to investigate whether diets containing individual SFA together with excess simple carbohydrates induce osteoarthritis (OA)-like changes in knee joints and signs of metabolic syndrome in rats. Rats were given either a corn starch diet or a diet composed of simple carbohydrates together with 20% LA, MA, PA, SA or beef tallow for 16 weeks. Rats fed beef tallow, SA, MA or PA diets developed signs of metabolic syndrome, and also exhibited cartilage degradation and subchondral bone changes similar to OA. In contrast, replacement of beef tallow with LA decreased signs of metabolic syndrome together with decreased cartilage degradation. Furthermore, PA and SA but not LA increased release of matrix sulphated proteoglycans in cultures of bovine cartilage explants or human chondrocytes. In conclusion, we have shown that longer-chain dietary SFA in rats induce both metabolic syndrome and OA-like knee changes. Thus, diets containing SFA are strongly relevant to the development or prevention of both OA and metabolic syndrome.

Obesity is a major risk factor for osteoarthritis (OA) and this has led to the description of metabolic syndrome-associated OA as a new OA phenotype^1^. In adipose tissue, adipocytes store lipids but they also produce and release many adipokines that are then responsible for the chronic low-grade inflammation in obesity^2^. Leptin, one of these adipokines, has been extensively studied in the pathophysiology of obesity^3^. Leptin may serve as the mechanism linking obesity and OA, because it is involved in cartilage and chondrocyte pathophysiology^4^. Increased intake of either mono- or poly-unsaturated fatty acids in a healthy population of middle aged adults was linked with increased possibility of developing bone marrow lesions^5^. Also, free fatty acids may be involved in inflammatory metabolic diseases such as obesity and inflammatory joint diseases^6^. Thus, dietary saturated fatty acids (SFA) could initiate the link between OA and obesity^7^,^8^,^9^. This is supported by the alterations in subchondral bone structure in patients with increased SFA intake^5^ that may lead to OA. Further, treatment of human chondrocytes with palmitate induced apoptosis and caused articular cartilage degradation^10^. However, there are no studies that have examined the association between these diseases using individual dietary SFA, despite the importance of dietary SFA as a potential initiator of chronic human diseases including obesity and OA.

Obesity is excessive fat storage in the body which is a major component of metabolic syndrome, a constellation of hypertension, diabetes, dyslipidaemia and fatty liver disease that increases the risk of cardiovascular disease^11^. Although, it has been widely accepted that SFA as a group promotes abdominal obesity, dyslipidaemia, insulin resistance, impaired glucose tolerance and systemic inflammation^12^, this role of SFA in obesity, diabetes and cardiovascular disease is now being challenged. Recent studies suggest that SFA-induced responses are dependent on chain length, with clear differences in biological responses between lauric acid and palmitic acid^13^. However, the roles of individual dietary SFA in OA have not been clearly defined.

Our previous studies reported that high-carbohydrate, high-fat diet (H; mainly containing fructose and beef tallow) for 16 weeks in young male Wistar rats mimics the symptoms of human metabolic syndrome including abdominal obesity, increased total body fat mass, elevated plasma lipids and blood pressure, impaired glucose tolerance and insulin sensitivity, fatty liver and cardiovascular remodelling^14^. The present study has investigated the role of C12 to C18 SFA (lauric acid (LA; C12:0), myristic acid (MA; C14:0), palmitic acid (PA; C16:0) and stearic acid (SA; C18:0) as well as beef tallow (mainly SA and *trans* fatty acids) on both the signs of metabolic syndrome and OA development.

## Results

### SFA on metabolic syndrome

Rats given a high-carbohydrate diet together with beef tallow containing both saturated and *trans* fats (H diet) developed changes associated with human metabolic syndrome including abdominal obesity, hyperleptinaemia, hyperlipidaemia and liver dysfunction compared to a corn starch diet (C diet) (Table 1), however, H rats did not show hyperglycaemia and hyperinsulinaemia compared to C rats. C diet has low energy density and possibly for this reason, the food intake was higher in C rats than all other groups. Although the food intake was higher in C rats, energy intakes were higher in all high-fat diet groups than in C rats in the order of C < (HLA = H) < HMA < (HPA = HSA) (Table 1). With lower body weight gain, the feed conversion efficiency was lower in HLA rats than in all other groups whereas H had higher feed conversion efficiency than other groups (Table 1). No difference in feed conversion efficiency was found in C, HMA, HPA and HSA although HMA, HPA and HSA had higher energy intakes. Body fat was highest in H rats and lowest in HLA rats while C, HMA, HPA and HSA had intermediate body fat. Lean mass was lower in HLA rats with no difference in other groups. Abdominal circumference was lower in HLA rats than in C rats while other groups were higher than C rats (Table 1). Abdominal fat pads were lowest in HLA rats while highest in H rats with other groups having intermediate abdominal fat. Basal blood glucose concentrations were lowest in HLA rats with HMA slightly higher than HLA. HSA and H had higher blood glucose concentrations than other groups while C and HPA had intermediate blood glucose concentrations. HLA showed higher plasma activities of alanine transaminase, aspartate transaminase and alkaline phosphatase than C rats. Alanine transaminase and alkaline phosphatase activities were higher in other groups than C rats whereas aspartate transaminase activity was equivalent to C rats. Plasma total cholesterol was higher in HLA rats than in C rats with other groups having intermediate concentrations of total cholesterol. Plasma NEFA was lowest in C rats with HLA NEFA higher than C rats and H group with highest plasma NEFA concentrations. Further, other groups had intermediate plasma NEFA concentrations. Plasma triglycerides were higher in HMA rats than C rats while HSA and H were further increased with H having the highest plasma triglycerides. Plasma insulin concentrations were lower in HLA rats than H, HMA, HPA and HSA with C rats having intermediate concentrations. Plasma leptin concentrations were highest in H rats while lowest in HLA rats (Table 1).

Rats given a high-carbohydrate diet together with beef tallow (H diet) showed increased systolic blood pressure and worsening cardiac function, both by echocardiography and also as an increased ventricular stiffness in isolated heart studies, without the development of heart failure, when compared to C rats. In contrast, HLA rats showed minor changes when compared with C rats. Replacement of beef tallow with HMA, HPA or HSA led to worsening cardiac function and increased systolic blood pressure compared with C or HLA rats (Table 1). Parameters such as relative wall thickness, fractional shortening, heart rate, LV developed pressure, ascending aorta flow and descending aorta flow did not show differences among the groups with different dietary fat sources (Supplementary Table 1).

### SFA on histological features of the articular cartilage

**Figure 1:**
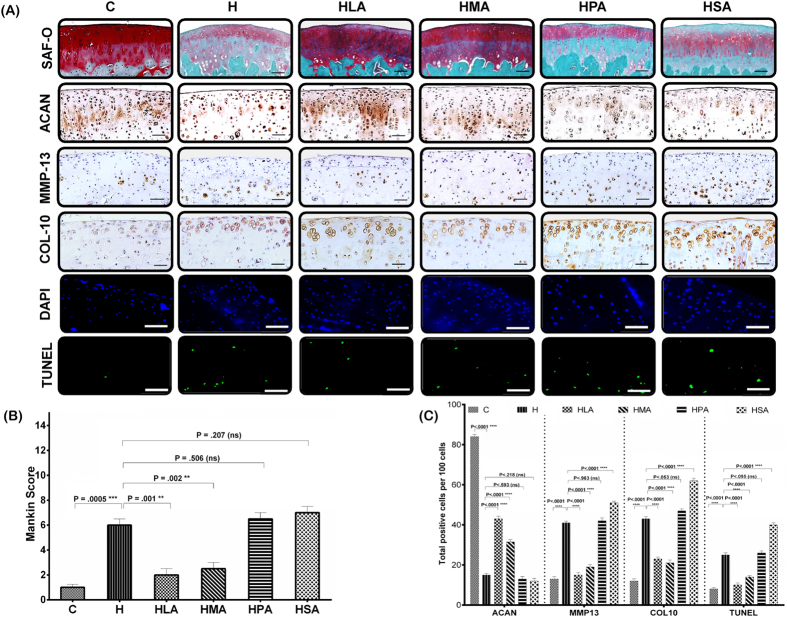
(**A**) Histological evaluation of the knee joints. All pictures were obtained under 20X magnification (n = 8 per group), representing the medial tibial plateau. Safranin O/Fast Green staining shows the extent of proteoglycan loss among the different diet groups. Representative immunohistochemical staining shows the number of positive cells for ACAN, MMP13, COL10 among the different diet groups. Analysis of apoptotic chondrocytes among different diets were quantified using TUNEL assay (DAPI and TUNEL images shown). The numbers of TUNEL-positive cells per section of the articular cartilage were determined under fluorescence microscopy. For each immunostaining, negative control either without primary antibody or with isotype-matched IgG instead of primary antibody was included. (**B**) Severity of articular cartilage degradation was graded using Mankin scoring system. (**C**) Quantitative histomorphometric analyses. Total positive cells per 100 cells in the cartilage were counted using ImageJ (NIH, Bethesda, MD). All values are represented as mean ± SD. Scale bar is 100 μm. (P < 0.05)

The joints from C rats showed smooth articular cartilage surface, normal cellularity and exhibited strong intensity of Safranin O staining. The chondrocytes were flattened in the tangential layer and were distributed in short irregular columns in the radial layer of the articular cartilage (Fig. 1A). Mankin scores were in the range of 0–1 in the C rats indicating normal cartilage structure (Fig. 1B). In contrast, H, HPA and HSA rats showed degeneration of the articular cartilage especially at the surface zone, indicated by surface irregularity, disorganisation of the articular cartilage with apparent chondrocyte clustering in the transitional and radial zones, and loss of proteoglycans shown by the weak intensity of Safranin O staining (Fig. 1A), with markedly increased Mankin scores (Fig. 1B). HMA and HLA rats showed cartilage structures that were similar to those of the C rats, with strong intensity of Safranin O staining and similar Mankin scores (Fig. 1A,B).

OA is characterised by reduced expression of the chondrogenic marker, aggrecan (ACAN), and increased expression of the hypertrophy and degradative markers, COL10 and MMP13. C rats showed strong expression of ACAN and limited expression of COL10 and MMP13. However, H, HPA and HSA rats exhibited degradative changes in cartilage structure that were greater than in HLA or HMA rats. ACAN expression decreased and expression of MMP13 and COL10 increased in H, HPA and HSA rats. HLA and HMA rats showed the least expression for MMP13 and COL10, but showed increased expression of ACAN in the cartilage compared to other SFA groups (Fig. 1C).

The number of apoptotic cells varied markedly between groups with only 8% of cells undergoing apoptosis in the C group compared to 24% in H rats. Replacement of beef tallow with HPA (26%) or HSA (40%) led to increased apoptosis that was greater than with HLA (10%) or HMA (14%) or in H rats (Fig. 1C).

### SFA on bone morphology

**Figure 2:**
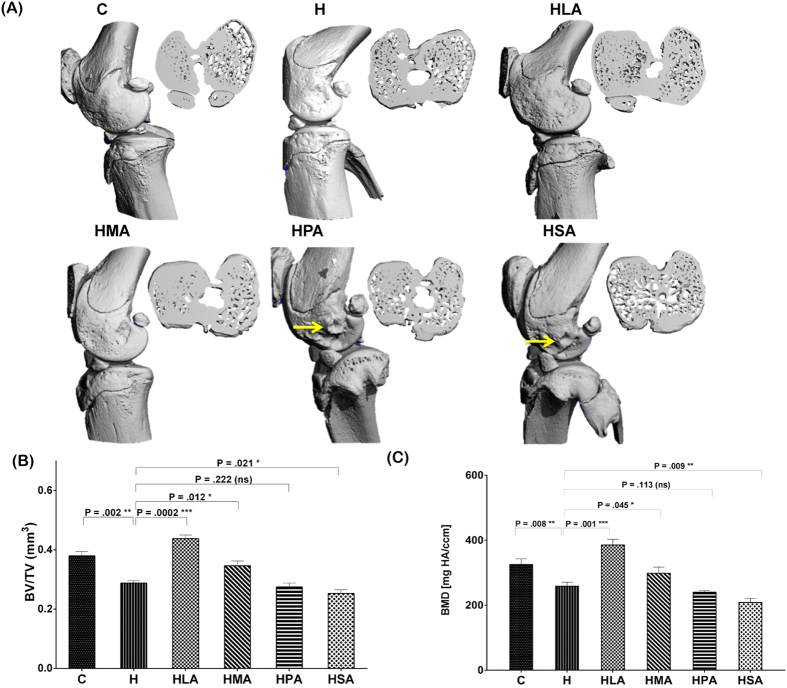
Micro-CT analyses of knee joints of rats fed the different diets (n = 8). (**A**) A 3-D Image of the whole knee joints of rats and the reconstructed axial micro CT cross-section images (insert) of region of interest i.e. the medial and lateral tibial plateau showing altered subchondral bone architecture (yellow arrows show abnormal bone morphological changes). For morphometric analyses, (**B**) the bone volume fraction (BV/TV) was calculated as the ratio of segmented bone volume (BV) to the total volume (TV) of the region of interest. In addition, (**C**) the bone mineral density (BMD) of the region of interest was also calculated. All values are represented as mean ± SD (P < 0.05).

Abnormal subchondral bone remodelling is recognised as a characteristic feature of OA. A 3-D representation of whole rat knee joint using micro-CT showed changes to the bone architecture of H, HPA and HSA rats compared to C, HLA or HMA rats (Fig. 2A). The region of interest was set as the medial and lateral tibial subchondral bone compartment. Tibial subchondral BV/TV decreased in H rats compared to that of the C rats. Replacement of beef tallow with LA or MA in diet increased tibial BV/TV while replacement of beef tallow with PA or SA decreased tibial BV/TV compared to H rats (Fig. 2B). Similarly to BV/TV, subchondral bone mineral density (BMD) decreased in rats fed with the beef tallow diet (H) compared to the C diet. Replacement of beef tallow with LA or MA increased BMD. In contrast, replacement of beef tallow with PA or SA in diet decreased BMD compared to the H rats (Fig. 2C).

### Diverse distribution of osteocytes in the subchondral bone region

In this study, the average osteocyte lacunae per mm^2^ (Avg.OS.L) was increased in H, HPA and HSA rats compared to C rats, suggesting increased bone remodelling and dysregulated mineral metabolism caused by the diets. Avg.OS.L density of HLA and HMA rats was very similar to that of the C rats. The decreased presence of average osteocyte nucleus (Avg. OS.N) in H, HMA, HPA and HSA rats compared to the C rats suggests the diets induced apoptotic death of the osteocytes (Supplementary Table 2).

### SFA on human chondrocytes and bovine cartilage explants

**Figure 3:**
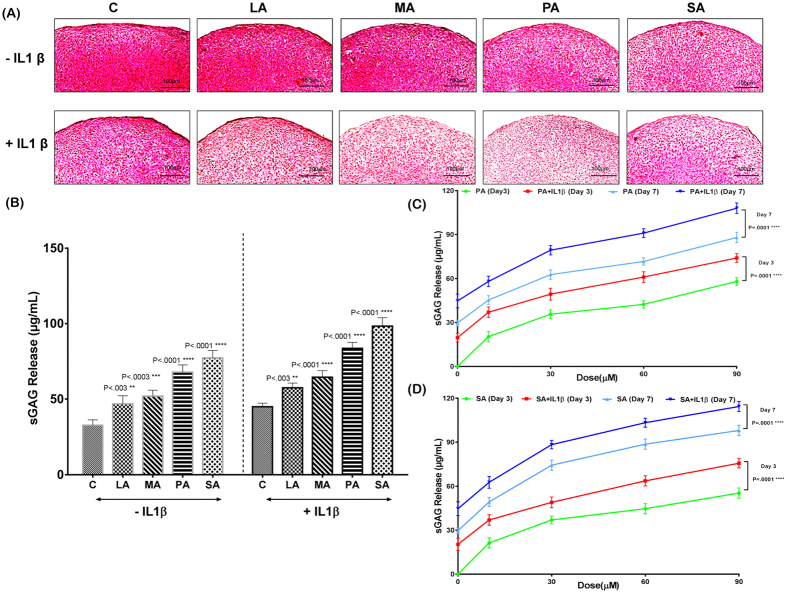
Proteoglycan content and sulphated glycosaminoglycan (sGAG) release of chondrocyte pellets stimulated with different SFA. All pictures were obtained under 20X magnification (n = 5 per group). (**A**) Safranin O/Fast Green staining shows the extent of proteoglycan loss among the different diet groups both in non-IL-1β treated and IL-1β treated pellets. (**B**) Quantification of sGAG release in the supernatant was performed by DMB assay, which showed that PA and SA-treated pellets exhibited a trend of decreased chondrogenesis irrespective of IL-1β treatment. (**C**,**D**) Graph showing the increase in sGAG release in response to increasing concentration of PA and SA. All values are represented as mean ± SD. Scale bar is 100 μm (P < 0.05).

Human chondrocytes isolated from cartilage specimens (grade 0–1) were stimulated with different SFA. To induce OA-like biological changes, the human chondrocytes were stimulated with IL-1β. There was an increased sGAG release from LA, MA and PA pellets compared to the control pellet at day 3. The greatest sGAG release was seen from the pellets treated with SA in comparison to the control pellets (Fig. 3B). On day 7, the control 3D cultured chondrocyte pellets treated with BSA (carrier) showed increased presence of proteoglycans indicated by strong Safranin O staining and decreased sGAG release into the media (Fig. 3A). LA and MA-treated pellets showed staining intensity similar to control pellets. In contrast, PA and SA-treated chondrocyte pellets showed marked loss of proteoglycans and subsequent increase in sGAG release into the medium compared with the other groups (Fig. 3A,B). Treatment with IL-1β alone decreased the proteoglycan content in the chondrocyte pellet compared to the non-IL1β treated control pellet, showing that IL-1β treatment promotes proteoglycan degradation. Addition of PA and SA increased the cartilage matrix degradation shown by decreased proteoglycan content in the chondrocyte pellet and increased release of sGAG into the medium compared to the control pellet, indicating the effect of IL-1β and SFA on cartilage degradation (Fig. 3A,B).

Since we observed increased sGAG release from both the PA and SA treated pellets, we performed a time course and a concentration-response experiment using these treatments. Increased concentrations of PA and SA increased release of sGAG into the medium. Once again, the IL-1β treated chondrocytes exhibited increased sGAG release compared to PA and SA pellets that were not treated with it (Fig. 3C,D). To assess if the concentration of IL-1β used in this study caused significant release of sGAG, we treated the chondrocyte pellets with different concentrations of IL-1β. The day 3 and day 7 sGAG release data showed that IL-1β at 10 ng/ml did not increase the rate of sGAG release in comparison with IL-1β at 1 ng/ml.

**Figure 4:**
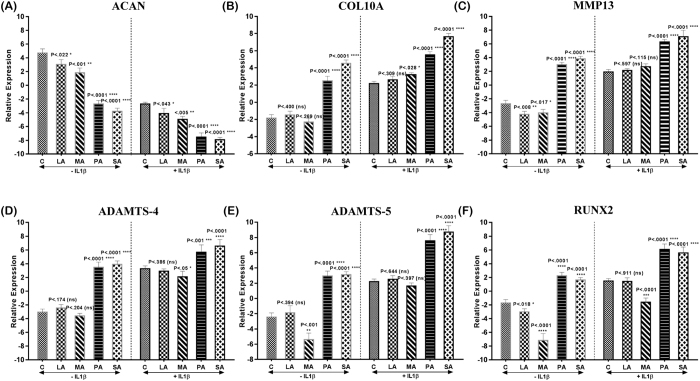
Extracellular matrix-related gene expression of chondrocyte pellets stimulated with different SFA (n = 5 per group). (**A**) ACAN, (**B**) COL10A, (**C**) MMP13, (**D**) ADAMTS4, (**E**) ADAMTS5 and (**F**) RUNX2 mRNA levels were assessed by RT-PCR both in non-IL-1β treated and IL-1β treated pellets. All experimental samples were performed in triplicate. All values are represented as mean ± SD (P < 0.05).

**Figure 5:**
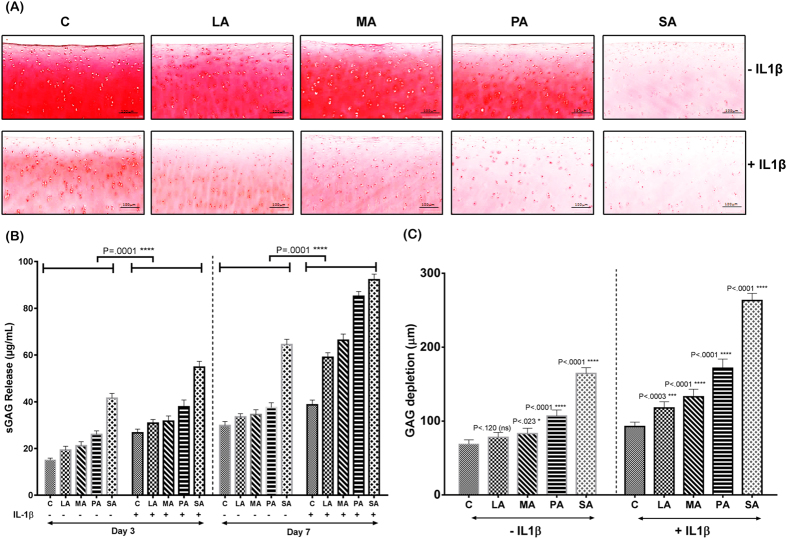
Proteoglycan content, sGAG release and sGAG depletion of bovine cartilage explants (n = 3) stimulated with different SFA. (**A**) Safranin O/Fast Green staining of bovine cartilage explants shows the loss of proteoglycans in the different diet groups both in non-IL-1β treated and IL-1β treated explants. (**B**) Quantification of sGAG release in the supernatant was performed by DMB assay, which showed that PA and SA-treated explants increased release of sGAG into the media on both day 3 and day 7 when compared to the other diet groups. In addition, zone of sGAG depletion (**C**) in the cartilage explants was the highest in SA-treated explants irrespective of IL-1β treatment (P < 0.05).

Loss of aggrecan (ACAN) is the first sign of degeneration with increased expression of degradative (MMP13, ADAMTS4, ADAMTS5) and hypertrophic markers (COL10 and RUNX2)^15^. Therefore, these markers were assessed in normal human chondrocytes treated with SFA with or without the addition of IL-1β using quantitative RT-PCR. In comparison with control samples, SFA-treated samples had decreased expression of ACAN (Fig. 4A) both in the presence and absence of IL-1β. The expression of ACAN was markedly down-regulated in LA and MA-treated groups, and the highest down-regulation was seen in PA and SA-treated groups both in the absence and presence of IL-1β. LA and MA-treated pellets had lower expression of COL10A (Fig. 4B), MMP13 (Fig. 4C), ADAMTS-4 (Fig. 4D), ADAMTS5 (Fig. 4E) and RUNX2 (Fig. 4F), in comparison with PA and SA-treated pellets (Fig. 5) in both the absence and presence of IL-1β.

The bovine explants in the control group treated only with bovine serum albumin (BSA) carrier exhibited a smooth articular cartilage surface and normal cellularity. Explants treated with LA or MA showed staining intensity similar to control explants. In contrast, explants treated with PA or SA showed decreased intensity of Safranin O staining compared to control explants (Fig. 5A). Consistent with the Safranin O staining results, marked increases in the release of sGAG were observed in the explants treated with PA or SA compared to control, LA or MA-treated explants (Fig. 5B). In bovine explants treated with IL-1β, the intensity of Safranin O staining was decreased upon stimulation of different SFA. Addition of LA and MA decreased the proteoglycan content of the cartilage explants compared to the control explant and also increased the release of sGAG into the medium. Furthermore, addition of PA and SA decreased the proteoglycan content of the cartilage explant compared to the control explant and also increased sGAG release into the medium (Fig. 5A,B). In addition, treatment of PA and SA increased the sGAG depletion compared to the control explants both in the absence and the presence of IL-1β. In contrast, treatment of LA and MA showed sGAG depletion which was comparable to that of the control treated explants. However, when IL-1β was added to the explants, LA and MA treatment increased the sGAG depletion of the explants compared to control explants, but were not lower compared to PA and SA treatment (Fig. 5C).

## Discussion

The worldwide incidence of obesity has doubled since 1980, now affecting 475–600 million people, depending on the BMI cut-off used in the estimates^16^. The incidence of OA has also markedly increased over a similar time period^17^, leading to well-documented support for the concept that obesity is a modifiable risk factor for the incidence and progression of OA^18^. The prevalence of both obesity and OA are greatest in patients over 65 years of age, and this is an increasing segment of the world’s population. Since both obesity and OA reduce mobility and increase cardiovascular risk, weight loss and exercise are recognised as key components for treatment of obese patients with OA^18^. Thus, understanding the common mediators for obesity and OA is clearly relevant to provide realistic and sustainable treatment options for obesity-associated OA.

Increased intake of dietary fat is one of the most important factors linking obesity and OA. Dietary fat is a key contributor to obesity and may help uncover the complex relationships between obesity and OA^19^. In obesity-related OA, SFA are relevant as they are the major dietary fatty acids. Our study strongly indicates that SFA produce parallel changes over the same time-frame in metabolic syndrome and typical OA-like lesions in the knee joint combined with increased chondrocyte death and breakdown of matrix in isolated bovine and human chondrocytes. Overall, the changes increase with SFA chain length with minimal changes following LA treatment to marked changes with SA treatment. These changes may be further increased by the presence of *trans* fatty acids as in beef tallow, but this proposal needs to be further tested by treatment with pure *trans* fatty acids. A plausible mechanism is suggested by the correlation between obesity and OA-like changes and plasma leptin concentrations but other hormones and cytokines released from macrophages may also be involved, as discussed below. This is consistent with the reported increase in palmitate- or stearic acid-induced apoptosis in chondrocytes^10^,^20^. In addition, previous studies have reported that SFA-rich high-fat diet increased the severity of OA^8^,^21^,^22^.

The changes in cartilage observed in this study are characteristic of OA-induced cartilage damage with increased PA and SA inducing marked depletion of proteoglycan content and increased expression of MMP13 and COL10 proteins shifting the balance of matrix homeostasis towards catabolism. MMPs are capable of degrading all components of the extracellular matrix, and their enhanced activity has been strongly implicated in cartilage degeneration^23^. Hypertrophy is known to trigger apoptosis of cartilage cells. The type of fatty acid clearly plays a role since LA did not induce these changes. PA and SA showed decreased proteoglycan content and increased release of sGAG, which confirmed their destructive properties on both cartilage explants and re-differentiated chondrocyte pellets. We further studied the effects of SFA and inflammatory cytokines such as IL-1β on chondrocytes. Co-treatment with IL-1β decreased the proteoglycan content and facilitated the increased release of sGAG in both human and bovine cartilage. Increases in gene expression of MMP13, ADAMTS 4 and 5 in PA- and SA-treated cartilage explants correlated with the increased release of sGAG. These results collectively suggest that catabolic effects of PA and SA together with IL-1β co-treatment in both human and bovine cartilage are likely to be mediated by the increased expression of cartilage-degrading enzymes that then trigger the changes similar to OA in chondrocytes. Furthermore, the number of empty lacunae is a clear indicator of osteocyte death^24^. In our recent study, the functional properties of osteocytes changed in OA patients, signifying the potential role of these cells in subchondral bone sclerosis^25^. Our results confirmed the increased presence of empty lacunae in H, HPA and HSA diet rats suggesting increased bone remodelling and unregulated mineral metabolism.

The correlation between obesity and OA is well-established, indicating that metabolic-induced inflammation in obesity^26^ plays an important role in OA^27^. The initiators of the inflammatory processes in rats fed this obesogenic diet are most likely to be SFA, since SFA such as PA activate macrophages^10^, and therefore increase infiltration of inflammatory cells throughout the body. Thus, obesity by itself does not cause OA, nor the reverse, but increased circulating SFA may induce infiltration of inflammatory cells throughout the body to produce parallel development of both disease states. While SFA may initiate the inflammation, there is good evidence that adipokines produced by inflammatory cells within fat pads also produce major physiological responses and may therefore increase the inflammatory responses in OA^28^. Key cytokines are likely to include leptin and adiponectin, with possible roles of more recently discovered adipokines such as resistin and visfatin, in both obesity and OA^4^,^28^,^29^. The involvement of these cytokines in the development of obesity-induced OA could underlie the improvements following loss of abdominal obesity^30^, and so decreased cytokine production, such as improved lean mass/fat mass ratio leading to increased mobility and physical activity with decreased OA-induced pain, for example in the knees. The local inflammatory reactions in obesity-associated OA may then be increased by the development of insulin resistance in type 2 diabetes^31^. Type 2 diabetes is strongly predictive of the severity of OA, independent of BMI and age^32^,^33^, suggesting the involvement of insulin as type 2 diabetes is an insulin-resistant state. Insulin decreased autophagy in immortalised human chondrocytes and human cartilage explants, providing a mechanism by which cartilage could be damaged in an insulin-resistant state^34^. The correlation of decreased plasma insulin concentrations and decreased OA-like changes in rats fed LA, and the increased OA-like changes in rats fed PA and SA in our study strongly supports the role of insulin in OA associated with glucose intolerance and obesity. Oxidative stress as an increased biological activity of oxygen free radicals is also a key initiator of damage in both obesity^35^ and OA^36^. Ageing is associated with an increased oxidative stress, and also increased prevalence of both obesity and OA, suggesting oxidative stress as a possible causative link. Further, functional foods such as omega-3 polyunsaturated fatty acids may improve oxidative status as an important therapeutic mechanism^37^.

The limitations of this study include that we have not measured the morphological changes of the synovium or changes in synovial fluid cytokine concentrations. These two additional parameters may contribute additional insights to understanding local changes due to inflammation in SFA diets and so improve understanding of obesity-induced OA.

In conclusion, this study provides evidence that SFA can produce similar changes in both metabolic syndrome and OA. These changes correlate with the plasma concentrations of leptin and insulin, both involved in obesity, type 2 diabetes and OA. Our data suggest that replacement of traditional diets containing coconut-derived LA with palm oil-derived PA or animal fat-derived SA has the potential to worsen the development of both metabolic syndrome and OA. Further, human clinical trials are necessary to determine whether replacement of PA and SA in the diet with LA will attenuate or reverse the development of both OA and metabolic syndrome, especially obesity and hypertension.

## Methods

### Study design and diets

All animal experiments for this study were approved by the Animal Ethics Committees of the University of Southern Queensland and Queensland University of Technology while the human sample collection was approved by the Prince Charles Hospital Human Ethics Committee. The procedures for using animal and human samples were performed in accordance with the guidelines of the National Health and Medical Research Council of Australia. The experimental groups consisted of 72 male Wistar rats (9–10 weeks old) weighing 330–350 g purchased from Animal Resource Centre, Perth, WA, Australia (www.arc.wa.gov.au). Rats were individually housed in a temperature-controlled, 12-hour light/dark cycle environment with free access to water and food at the University of Southern Queensland Animal House. The rats were randomly divided into 6 groups of 12 rats. Corn starch (C) diet contained 57% corn starch, 15.5% powdered rat food (Specialty Feeds, Glen Forest, WA, Australia), 2.5% HMW salt mixture and 25% water. High-carbohydrate, high-fat diet consisted of 17.5% fructose, 39.5% sweetened condensed milk, 20% beef tallow (H) or SFA (20% LA (HLA), 20% MA (HMA), 20% PA (HPA) or 20% SA (HSA)), 15.5% powdered rat food, 2.5% HMW salt mixture and 5% water. C diet included drinking water without any additives while H-based diet-fed rats were given drinking water supplemented with 25% fructose^14^. The carbohydrate intake in both C and H groups was approximately 68%. Rats had *ad libitum* access to food and water during the protocol.

### Measurements of metabolic variables

Daily measurements of body weight and intakes of food and water were performed to monitor the day-to-day health of rats. Feed conversion efficiency (%) was calculated as previously described^14^. Percent body weight increase over 16 weeks was calculated as body weight difference between day 0 and day 112. Abdominal circumference was measured every 4 weeks using a standard measuring tape under light anaesthesia with Zoletil (tiletamine 10 mg/kg, zolazepam 10 mg/kg i.p; Virbac, Peakhurst, NSW, Australia).

Oral glucose tolerance tests were performed on rats as previously described^14^. Briefly, rats were deprived of food for 12 hours before basal blood glucose concentration measurements followed by oral gavage of 40% aqueous glucose solution and measuring glucose concentrations again at 30, 60, 90 and 120 minutes, with calculation of AUC (area under the curve) from these measurements. Systolic blood pressure was measured every 4 weeks under light sedation with Zoletil (10 mg/kg tiletamine, 10 mg/kg zolazepam, i.p.)^14^. Echocardiography was performed to measure the cardiovascular structure and function^14^. Indirect calorimetry was used to measure oxygen consumption and carbon dioxide production using a 4-chamber Oxymax system (Columbus Instruments, Columbus, OH) with one rat per chamber. Rats had *ad libitum* access to food and water during the measurement. Oxygen consumption (V_O2_) and carbon dioxide production (V_CO2_) were measured individually from each chamber. The respiratory exchange ratio (RER = V_CO2_/V_O2_) was calculated by Oxymax software (v. 4.86). The oxidation of carbohydrates produces an RER of 1.00, whereas fatty acid oxidation results in an RER of about 0.70^38^. Energy expenditure was calculated by assessment of the exchange of oxygen for carbon dioxide that occurs during the metabolic processing of food.

Rats were euthanised after 16 weeks using Lethabarb^®^ (100 mg/kg pentobarbitone sodium, i.p.). After euthanasia, blood was collected to isolate plasma and the plasma was stored at −20 °C before further analysis. Hearts were isolated to perform Langendorff heart preparation to measure diastolic stiffness constant^14^. Following this, tissues such as liver, left ventricle (with septum), right ventricle and abdominal fat pads (including retroperitoneal, epididymal and omental) were removed for weighing and expressed as mg/mm of tibial length. Plasma concentrations of leptin, insulin, total cholesterol, triglycerides and non-esterified fatty acids (NEFA) were measured as described previously^14^.

### Assessment of articular cartilage

After 16 weeks of dietary interventions, rats were euthanised and the knee joints harvested and then processed for histology. Briefly, the intact joints were fixed in 4% paraformaldehyde for 48 hours, decalcified in 10% EDTA for 8 weeks, bisected laterally and embedded in paraffin. Paraffin blocks were then trimmed to expose tissue using a rotary microtome (Leica, Wetzlar, Germany). Two 5-μm sections within every consecutive six sections were cut and mounted on glass slides. Each knee yielded about 12 sections for standard haematoxylin and eosin (H&E) staining or proteoglycan staining with safranin O as previously described^39^. The severity of cartilage damage was assessed using the standard Mankin grading system^40^. The mean damage score from 5–6 samples from each animal was used to determine the mean ± standard deviation for each group. Two independent observers assessed cartilage damage in a blinded manner. Furthermore, immunostaining was performed with antibodies against matrix metalloproteinase-13 (MMP13) (1:50 Labvison, RB1681-P0, Fremont, CA, USA), aggrecan (AGAN) (1:200 Millipore, AB1031, New South Wales, Australia) and type 10 collagen (COL10) (1:200 Abcam, ab58632, New South Wales, Australia)^39^. For controls, the same procedures were carried out either without primary antibody or with isotype-matched IgG instead of primary antibody. For semi-quantitative data analysis, the positive cells from different fields of observation in the tibial medial compartment of the knee were counted and normalised to the cell number per 100 total cells in each group, using ImageJ (NIH, Bethesda, MD)^39^.

### TUNEL apoptosis assay

Terminal deoxynucleotidyl transferase (TdT) dUTP Nick-End Labelling (TUNEL) was used to identify cells that show degradation of their DNA during apoptosis. The tissue slices were first permeabilised with proteinase K for 30 minutes at 37 °C. The slides were then washed with PBS and the assay was performed using the manufacturer’s protocol (Roche, Germany). The sections were incubated with DNase1 for 10 minutes at room temperature as a positive control. For semi-quantitative data analysis, the positive cells from different fields of observation were counted and normalised to the cell number per 100 total cells in each group, using ImageJ (NIH, Bethesda, MD)^39^.

### Assessment of subchondral bone changes

Micro-CT was performed to analyse the subchondral bone changes. Femur and tibia were scanned using a micro-CT (Scanco μCT 40, Scanco Medical, Switzerland) with isotropic voxel size of 18 μm with voltage of 55 kV and current of 145 μA with a 0.5 mm aluminium filter. The exposure time was 1180ms and Scanco’s inbuilt software was used to segment the data set^39^. Manual regions of interest (ROI) were drawn around the anatomical contour in the subchondral bone region in the medial and lateral tibial plateau. The volume of interest (VOI) consisted of a stack of ROIs (25 cross-sections). The VOI started below the subchondral plate which extended distally towards the growth plate. Ratio of bone volume to the total volume (BV/TV) and bone mineral density (BMD) of the VOI were calculated.

### Analysis of osteocytes

Average osteocyte empty lacunae (Avg OS.L) and average osteocyte nucleus (Avg OS.N) were counted per unit area (mm^2^) in the subchondral bone region in the medial tibial plateau using ImageJ (NIH, Bethesda, MD).

### Treatment of chondrocytes and cartilage explants with SFA

#### Preparation of SFA

LA, MA, PA and SA were purchased commercially at the highest purity available (Sigma-Aldrich, GC-purified, chemically synthesised). Bovine serum albumin (BSA) (fatty-acid free, Sigma Aldrich) was chosen as the carrier. The SFA-BSA complex solutions were made using a protocol described earlier^6^. Individual SFA were dissolved in 100% ethanol at 70 °C to yield a 200 mM stock solution. The stock solution was then diluted 1:10 in 10% (w/v) BSA made with Dulbecco’s Modified Eagle Medium (DMEM) for 10 minutes at 55 °C. The resultant SFA solution (20 mM) was sterile-filtered (0.45 μM filter) prior to application to the cell cultures using a protocol described earlier^6^. The vehicle control (negative control) was the fatty acid free-BSA with the same concentration of ethanol.

#### Chondrocyte pellet and bovine explant culture

Chondrocytes were harvested using enzymatic digestion and cultured using our published protocols^39^. Briefly, biopsies of articular cartilage were obtained from primary OA patients undergoing knee replacement surgery at The Prince Charles Hospital (Brisbane, QLD, Australia). All cartilage biopsies were taken from a part of the surface of the femoral condyle considered by the surgeon to resemble intact and healthy cartilage. The five donors were all males aged 60–65 years and all provided written informed consent. The study was approved by The Prince Charles Hospital and Queensland University of Technology Human Ethics Committees. Each cartilage specimen was further characterised and scored according to Mankin score with only samples with scores of 0–1 (healthy cartilage) being used for further experiments.

The cartilage was dissected from the bone and digested with collagenase 2 solution (Invitrogen, Lakewood, NJ) overnight in the DMEM culture medium for the isolation of the articular cartilage chondrocytes (ACC). After digestion, ACCs (2.5 × 10^5^ cells) were pelleted by centrifugation in 15 mL Falcon tubes and cultured in three-dimensional (3D) pellet culture system^39^, for 14 days in chondrogenic medium in the presence or absence of different SFA.

For bovine explant studies, fresh adult bovine knee joints (n = 3) were obtained on the day of slaughter. Full thickness articular cartilage explants without the subchondral bone were then harvested from the knee joints. Cartilage discs were then aseptically dissected using a 4-mm dermal punch. The discs were cultured in DMEM, and supplemented with 10% Foetal Bovine Serum (FBS) and antibiotics at 37 °C with 5% CO_2_ for 48 hours.

The pellets and discs were then stimulated with each SFA (final concentration: 30 μg/ml, derived from MTT assay – data not shown). The negative control, in which no SFA were added, was treated with BSA vehicle only. To study the effects of SFA and inflammatory cytokines, some ACC pellets and bovine cartilage discs were treated with IL-1β (10 ng/mL) in the presence or absence of different SFA. After day 3 and day 7, media were collected and stored in −80 °C for quantification of sulphated glycosaminoglycans. The cartilage discs and ACC pellets were later processed for histological analysis and quantitative real time PCR analysis (qPCR). To further assess the responses to certain SFA, different concentrations (10 μM, 30 μM, 60 μM and 90 μM) of PA and SA were added to chondrocyte pellets both in the absence and in the presence of IL-1β (10 ng/mL). After day 3 and day 7, media were collected and stored at −80 °C for quantification of sulphated glycosaminoglycans (sGAG). In addition, to assess the effects of IL-1β used in this study, chondrocyte pellets were treated with various concentrations of IL-1β (1–10 ng/ml). The day 3 and day 7 media were collected and stored at −80 °C for quantification of sGAG.

#### Sulfated glycosaminoglycans (sGAG) assay

To measure sGAG release, supernatants were collected from the wells containing the bovine cartilage discs or chondrocyte pellets treated with or without SFA at day 3 and day 7. The sGAG was measured using the dimethylmethylene blue method (DMB) with the kit (Blyscan™, Biocolor Ltd) following the manufacturer’s instructions. Zone of sGAG depletion in cartilage explants were determined using images of Safranin O staining and were measured using ImageJ (NIH, Bethesda, MD).

#### RNA extraction and real-time PCR

Total RNA was extracted using TRIzol reagent (Invitrogen), treated with DNase and purified according to manufacturer’s protocol using an RNeasy Mini Kit (Qiagen). cDNA was synthesised from 1 μg of the total RNA according to manufacturer’s protocol using an SensiFAST cDNA Synthesis Kit. Real-time quantitative PCR^41^, using SYBR Green detection chemistry, was performed on the ABI 7500 Fast Real Time PCR system (Applied Biosystems, Foster City, CA, USA). Melt curve analyses of all real-time PCR products were performed and shown to produce a single DNA duplex. All samples were measured in triplicate and the mean value of all experimental samples was considered for comparative analysis. Quantitative measurements of all primers used in this study were determined using the (2^−ΔΔ*Ct*^) method, and 18 s and β-actin expression were used as the internal controls, as described previously by our group^39^,^41^,^42^,^43^.

#### Statistics

Statistical analyses were performed using Graphpad Prism. The data are presented as mean ± standard deviation (SD) for all variables and analysed with ANOVA method. Repeated-measures analysis of variance with *post hoc* tests (Dunnett’s/Bonferroni) was used to assess statistical significance. The level of significance was set at P < 0.05.

## Additional Information

**How to cite this article**: Sekar, S. *et al*. Saturated fatty acids induce development of both metabolic syndrome and osteoarthritis in rats. *Sci. Rep.* **7**, 46457; doi: 10.1038/srep46457 (2017).

**Publisher's note:** Springer Nature remains neutral with regard to jurisdictional claims in published maps and institutional affiliations.

## Supplementary Material

Supplementary Tables

## Figures and Tables

**Table 1:** Dietary intake and body composition in C, H, HLA, HMA, HPA and HSA rats.

Variables	C	HLA	HMA	HPA	HSA	H
Food intake (*g/d)*	40.2 ± 2.2^a^	24.2 ± 3.3^d^	27.7 ± 1.3^c^	29.7 ± 2.6^bc^	30.1 ± 2.4^b^	25.0 ± 2.3^d^
Water intake (*mL/d)*	26.1 ± 4.1^c^	36.5 ± 4.5^a^	29.1 ± 2.4^b^	34.4 ± 2.2^a^	35.1 ± 2.7^a^	25.2 ± 3.5^c^
Body weight gained (*%)*	26.6 ± 6.3^c^	−11.1 ± 9.8^d^	34.7 ± 6.8^bc^	42.1 ± 15.3^b^	34.1 ± 11.9^bc^	58.6 ± 14.1^a^
Energy intake (*kJ/d)*	452 ± 24^d^	545 ± 70^c^	601 ± 26^b^	652 ± 46^a^	671 ± 52^a^	543 ± 44^c^
Feed conversion efficiency (*%)*	19.3 ± 4.1^b^	−7.4 ± 6.5^c^	19.3 ± 3.5^b^	21.1 ± 6.2^b^	17.9 ± 4.4^b^	36.2 ± 6.8^a^
Total body fat mass (*g)*	79.5 ± 34.7^cd^	54.4 ± 9.7^d^	132.4 ± 31.2^b^	122.7 ± 40.9^bc^	122.9 ± 30.0^bc^	207.7 ± 81.6^a^
Total body lean mass (*g)*	305.0 ± 23.6^a^	229.3 ± 19.5^b^	300.3 ± 23.0^a^	305.9 ± 25.9^a^	307.1 ± 23.6^a^	310.6 ± 46.9^a^
Abdominal circumference (*cm)*	20.2 ± 0.7^b^	17.4 ± 1.1^c^	21.5 ± 0.9^a^	21.5 ± 0.8^a^	21.4 ± 1.0^a^	22.4 ± 1.1^a^
Visceral adiposity index (*%)*	4.01 ± 0.88^c^	3.56 ± 0.96^c^	5.93 ± 0.80^b^	5.35 ± 1.45^bc^	4.94 ± 1.26^bc^	10.07 ± 2.59^a^
RER	1.01 ± 0.06^a^	0.90 ± 0.08^a^	0.94 ± 0.06^a^	1.01 ± 0.05^a^	1.01 ± 0.09^a^	1.01 ± 0.07^a^
Heat (*Kcal)*	3.16 ± 0.38^b^	2.46 ± 0.24^c^	3.33 ± 0.41^b^	3.47 ± 0.35^ab^	3.44 ± 0.29^ab^	3.87 ± 0.23^a^
Retroperitoneal fat (*mg/mm)*	155.2 ± 46.7^bc^	98.0 ± 36.5^c^	256.3 ± 48.6^b^	248.4 ± 92.1^b^	205.6 ± 53.2^bc^	572.2 ± 219.1^a^
Epididymal fat (*mg/mm)*	98.0 ± 21.0^b^	49.0 ± 14.0^c^	128.6 ± 22.7^b^	128.7 ± 63.0^b^	120.3 ± 41.7^b^	273.9 ± 69.5^a^
Omental fat (*mg/mm)*	88.7 ± 26.8^cd^	66.6 ± 18.1^d^	153.1 ± 22.3^b^	138.2 ± 51.5^b^	126.9 ± 30.9^bc^	257.9 ± 73.9^a^
Total abdominal fat (*mg/mm)*	342 ± 85^cd^	214 ± 66^d^	538 ± 84^b^	515 ± 201^b^	453 ± 116^bc^	1017 ± 255^a^
Basal blood glucose concentrations (*mmol/L*)	3.59 ± 0.30^ab^	2.64 ± 0.46^c^	3.16 ± 0.37^b^	3.46 ± 0.56^ab^	3.98 ± 0.81^a^	3.86 ± 0.51^a^
Systolic blood pressure (*mmHg)*	126.9 ± 5.5^d^	136.2 ± 16.9^c^	141.8 ± 3.7^bc^	150.4 ± 7.9^ab^	152.9 ± 11.0^a^	157.7 ± 10.7^a^
**Hepatic Function**						
Liver (*mg/mm)*	203.2 ± 28.0^c^	201.0 ± 27.6^c^	303.8 ± 24.8^b^	322.2 ± 49.5^ab^	295.8 ± 33.9^b^	345.9 ± 33.6^a^
Alanine transaminase (*U/L)*	19.4 ± 5.4^c^	54.2 ± 12.5^a^	47.3 ± 9.6^ab^	37.7 ± 7.6^b^	39.0 ± 8.9^b^	40.9 ± 11.1^b^
Aspartate transaminase (*U/L)*	57.4 ± 13.4^b^	75.3 ± 13.3^a^	60.4 ± 11.6^b^	56.2 ± 5.1^b^	55.5 ± 8.7^b^	66.4 ± 7.8^ab^
Alkaline phosphatase (*U/L)*	121.7 ± 24.8^c^	326.1 ± 66.0^a^	339.8 ± 80.2^a^	234.9 ± 63.7^b^	213.3 ± 37.6^b^	248.7 ± 59.5^b^
Total cholesterol (*mmol/L)*	1.42 ± 0.36^c^	1.98 ± 0.26^a^	1.78 ± 0.29^ab^	1.60 ± 0.18^bc^	1.69 ± 0.41^abc^	1.59 ± 0.11^bc^
NEFA (*mmol/L)*	1.09 ± 0.37^d^	2.32 ± 1.16^c^	3.15 ± 1.05^bc^	3.63 ± 1.69^ab^	4.10 ± 1.55^ab^	4.84 ± 1.13^a^
Triglycerides (*mmol/L)*	0.36 ± 0.17^d^	0.67 ± 0.42^cd^	0.88 ± 0.33^c^	1.04 ± 0.46^bc^	1.42 ± 0.68^b^	1.92 ± 0.67^a^
**Plasma hormone concentrations**						
Insulin (*μg/L)*	2.35 ± 1.80^ab^	1.42 ± 1.22^b^	3.81 ± 2.25^a^	3.60 ± 1.23^a^	3.59 ± 2.15^a^	4.12 ± 1.09^a^
Leptin (*μg/L)*	3.21 ± 1.12 ^cd^	1.93 ± 1.05^d^	7.94 ± 2.35^bc^	5.54 ± 3.71^c^	9.67 ± 3.87^ab^	11.37 ± 3.80^a^

Measurement of dietary intake and body composition in rats fed the different diets (n = 10–12). All values are represented as mean ± SD. Mean values within a row with unlike superscript letters are significantly different (P < 0.05) with a > b > c > d.

Abbreviations: C – corn starch diet-fed rats; H – high-carbohydrate, high-fat diet-fed rats; HLA – high-carbohydrate, high-lauric acid-fed rats; HMA – high-carbohydrate, high-myristic acid-fed rats; HPA – high-carbohydrate, high-palmitic acid-fed rats; HSA – high-carbohydrate, high-stearic acid-fed rats; RER – respiratory exchange ratio; NEFA – non-esterified fatty acids.
