# Supplementary material for: Saturated fatty acids induce development of both metabolic syndrome and osteoarthritis in rats
Source: Sci Rep. 2017 Apr 18;7:46457. doi: 10.1038/srep46457 (PMC5394476; doi:10.1038/srep46457)
Supplement: Supplementary Tables [file srep46457-s1.pdf]

Scientific Reports revised (Supplementary data)

Saturated fatty acids induce development of both metabolic syndrome and osteoarthritis in rats

Sunderajhan Sekar[1], Siti Raihanah Shafie[2], Indira Prasadam[1], Ross Crawford[3], Sunil K. Panchal[2], Lindsay Brown[2], Yin Xiao[1]

1. Institute of Health and Biomedical Innovation, School of Chemistry, Physics, Mechanical Engineering, Queensland University of Technology, Brisbane, Australia
2. Institute for Agriculture and the Environment, and School of Health and Wellbeing, University of Southern Queensland, Toowoomba, Queensland, Australia
3. The Prince Charles Hospital, Orthopaedic Department, Brisbane, Australia

Key words: Osteoarthritis, Saturated fatty acids, Obesity, Diet, Cartilage

Corresponding Author:

Professor Yin Xiao

Institute of Health and Biomedical Innovation, Queensland University of Technology, Kelvin Grove Campus, Brisbane, QLD 4059, Australia

Tel: + 61 7 3138 6240; Fax: + 617 3138 6030; Email: yin.xiao@qut.edu.au

Supplementary Table 1: Cardiovascular function in C, H, HLA, HMA, HPA and HSA rats

Variable	C	HLA	HMA	HPA	HSA	H
Heart weight (*mg/mm)*	21.8 ± 2.5[b]	16.2 ± 1.5[c]	23.7 ± 2.2[ab]	25.9 ± 4.1[a]	22.3 ± 2.3[b]	25.3 ± 3.0[a]
LV + septum wet weight (*mg/mm)*	18.2 ± 2.3[a]	13.0 ± 1.2[b]	19.3 ± 1.7[a]	20.1 ± 3.1[a]	18.2 ± 2.1[a]	20.0 ± 2.7[a]
RV wet weight (*mg/mm)*	3.61 ± 0.90[cd]	3.11 ± 0.38[d]	4.38 ± 0.79[bc]	5.78 ± 1.36[a]	4.08 ± 0.39[bc]	4.72 ± 0.76[b]
LVIDd (*mm)*	7.36 ± 0.61[b]	6.52 ± 0.52[c]	7.55 ± 0.73[b]	7.93 ± 0.51[ab]	7.86 ± 0.56[ab]	8.33 ± 0.46[a]
LVPWd (*mm)*	1.65 ± 0.25[ab]	1.55 ± 0.13[b]	1.71 ± 0.11[ab]	1.83 ± 0.11[a]	1.72 ± 0.18[ab]	1.79 ± 0.16[a]
Relative wall thickness	0.46 ± 0.10[a]	0.49 ± 0.06[a]	0.46 ± 0.04[a]	0.47 ± 0.04[a]	0.45 ± 0.06[a]	0.43 ± 0.05[a]
Fractional shortening (*%)*	53.2 ± 10.5[a]	50.5 ± 15.4[a]	50.4 ± 9.4[a]	42.6 ± 7.5[a]	41.9 ± 6.2[a]	43.0 ± 5.2[a]
Heart rate (*bpm)*	343 ± 95[a]	296 ± 78[a]	360 ± 48[a]	329 ± 81[a]	310 ± 74[a]	323 ± 54[a]
Stroke volume (μL)	375 ± 96[b]	246 ± 47[c]	391 ± 92[b]	420 ± 72[ab]	412 ± 99[ab]	493 ± 77[a]
Cardiac output (μL)	133.5 ± 61.1[a]	71.3 ± 18.1[b]	139.1 ± 32.8[a]	137.2 ± 38.1[a]	125.1 ± 34.7[a]	159.9 ± 40.3[a]
Diastolic stiffness constant (κ)	22.0 ± 1.4[c]	21.8 ± 2.9[c]	25.3 ± 2.2[b]	26.6 ± 2.2[ab]	27.1 ± 1.3[ab]	28.2 ± 1.5[a]
Estimated LV mass, Litwin (*g)*	0.83 ± 0.08[cd]	0.67 ± 0.08[d]	0.89 ± 0.16[bc]	1.03 ± 0.11[ab]	0.96 ± 0.11[ab]	1.06 ± 0.13[a]
Systolic wall stress ($mmHg.cm^{-1}$)	75.8 ± 28.7[b]	101.7 ± 58.2[ab]	95.0 ± 35.8[ab]	127.9 ± 35.3[a]	137.8 ± 26.6[a]	131.7 ± 28.6[a]
LV developed pressure (*mmHg)*	69.7 ± 22.3[a]	74.0 ± 29.6[a]	53.1 ± 16.9[a]	84.4 ± 31.0[a]	78.1 ± 27.0[a]	66.6 ± 30.5[a]
Ascending aorta flow (c*m/sec)*	98.6 ± 19.1[a]	83.4 ± 21.4[a]	96.3 ± 12.4[a]	97.8 ± 11.0[a]	98.9 ± 8.6[a]	97.4 ± 14.6[a]
Descending aorta flow (c*m/sec)*	87.3 ± 17.2[a]	81.4 ± 12.8[a]	95.1 ± 9.1[a]	97.0 ± 18.6[a]	95.0 ± 18.8[a]	96.2 ± 18.4[a]
Ejection time (*msec)*	78.2 ± 8.1[b]	94.9 ± 9.0[a]	80.4 ± 9.1[b]	84.0 ± 7.5[b]	85.6 ± 12.2[ab]	87.0 ± 7.2[ab]

Supplementary table 1 Legend: Measurement of cardiovascular function in rats fed the different diets (n=10). All values are represented as mean ± SD. Mean values within a row with unlike superscript letters are significantly different ($P < 0.05$) with a>b>c>d.

Abbreviations: C – corn starch diet-fed rats; H – high-carbohydrate, high-fat diet-fed rats; HLA – high-carbohydrate, high-lauric acid-fed rats; HMA – high-carbohydrate, high-myristic acid-fed rats; HPA – high-carbohydrate, high-palmitic acid-fed rats; HSA – high-carbohydrate, high-stearic acid-fed rats; LV – left ventricle; RV – right ventricle; LVIDd – left ventricular internal diameter during diastole; LVPWd – left ventricular posterior wall thickness during diastole.

Supplementary Table 2: Osteocyte morphology in C, H, HLA, HMA, HPA and HSA rats

**Variables**	**C**	**HLA**	**HMA**	**HPA**	**HSA**	**H**
**Avg OS.L/mm^2**	67.5±17.08	65.5±21.60	70.5±17.08	122.5±21.60[a]	182.5±18.93[a]	162.5±19.15[a]
**Avg OS.N/mm^2**	712.5±50.58	660.0±63.77	525.0±42.03[a]	460.0±35.59[a]	420.0±29.44[a]	512.5±34.03[a]
**Total Osteocyte Lacunae/mm^2**	787.5±37.75	727.5±50.58	597.5±65.51[a]	585.0±58.02[a]	587.5±56.79[a]	657.5±59.09[a]

Supplementary table 2 Legend: Diverse distribution of osteocytes in the subchondral bone region of the rats fed the different diets (n=8). The rats fed with H, PA and SA had increased Avg OS.L and decreased Avg OS.N compared to other diet groups. Values with superscript letter (a) are significantly different ($P<.05$). All values are represented as mean ± SD.
